# Transcriptome, carbohydrate, and phytohormone analysis of *Petunia hybrida* reveals a complex disturbance of plant functional integrity under mild chilling stress

**DOI:** 10.3389/fpls.2015.00583

**Published:** 2015-07-28

**Authors:** Martin Andreas Bauerfeind, Traud Winkelmann, Philipp Franken, Uwe Druege

**Affiliations:** ^1^Department of Plant Propagation, Leibniz Institute of Vegetable and Ornamental CropsErfurt, Germany; ^2^Institute of Horticultural Production Systems, Leibniz Universität HannoverHannover, Germany

**Keywords:** *Petunia*, cold, carbohydrate metabolism, sugars, invertase, gene expression, microarray, plant hormones

## Abstract

Cultivation of chilling-tolerant ornamental crops at lower temperature could reduce the energy demands of heated greenhouses. To provide a better understanding of how sub-optimal temperatures (12°C vs. 16°C) affect growth of the sensitive *Petunia hybrida* cultivar ‘SweetSunshine Williams’, the transcriptome, carbohydrate metabolism, and phytohormone homeostasis were monitored in aerial plant parts over 4 weeks by use of a microarray, enzymatic assays and GC-MS/MS. The data revealed three consecutive phases of chilling response. The first days were marked by a strong accumulation of sugars, particularly in source leaves, preferential up-regulation of genes in the same tissue and down-regulation of several genes in the shoot apex, especially those involved in the abiotic stress response. The midterm phase featured a partial normalization of carbohydrate levels and gene expression. After 3 weeks of chilling exposure, a new stabilized balance was established. Reduced hexose levels in the shoot apex, reduced ratios of sugar levels between the apex and source leaves and a higher apical sucrose/hexose ratio, associated with decreased activity and expression of cell wall invertase, indicate that prolonged chilling induced sugar accumulation in source leaves at the expense of reduced sugar transport to and reduced sucrose utilization in the shoot. This was associated with reduced levels of indole-3-acetic acid and abscisic acid in the apex and high numbers of differentially, particularly up-regulated genes, especially in the source leaves, including those regulating histones, ethylene action, transcription factors, and a jasmonate-ZIM-domain protein. Transcripts of one Jumonji C domain containing protein and one expansin accumulated in source leaves throughout the chilling period. The results reveal a dynamic and complex disturbance of plant function in response to mild chilling, opening new perspectives for the comparative analysis of differently tolerant cultivars.

## Introduction

Petunia (*Petunia hybrida*) is one of the economically most important ornamental crops in Europe and North America. In Germany, it is one of the top ten bedding plant species. In 2012, German ornamental crop-producing companies produced more than 43 million petunias (Bundesministerium für Ernährung, Verbraucherschutz und Landwirtschaft, 2013). In moderate and northern climates, bedding crops like petunia are mainly produced during the winter months and early spring. Thus, heating of greenhouses is crucial to maintain optimal conditions for production of these thermophile plants. Exemplary, for greenhouse cultivation of tomato, Elings et al. (2005) reported energy savings of 16%, obtained from a 2 K reduction of mean cultivating temperature. Consequently, production at lower temperatures could save a lot of energy and significantly reduce emission of greenhouse gases, contributing to a more sustainable production. However, production at sub-optimal temperature would lead to retarded plant growth and development, which would extend production time and thus increase energy consumption again. For that reason, cultivars are needed which show a minimum growth depression when cultivated at reduced temperature compared to optimal conditions. Better understanding of how mild chilling stress in response to slightly reduced temperatures affects plant growth would ease the breeding of such cultivars. In the context of the presented research, mild chilling is understood as a slight reduction of temperature below commonly used production temperatures, which provokes a significant decrease in growth without causing visible cold damage.

Previous research covered cold and freezing reactions of *Arabidopsis thaliana* (Zhu et al., 2007; Guy et al., 2008; Shi et al., 2012) and also of other plant species (Sasaki et al., 2001; Zhang et al., 2012). However, only few studies have addressed plant responses to mild chilling at slightly reduced temperatures (Usadel et al., 2008; Yamori et al., 2009). Although *Arabidopsis* is the most advanced and best investigated model plant species, it does not perfectly represent all other species (Schwarz-Sommer et al., 2003). The genus *Petunia* serves as outstanding model system regarding ecological niches and diversity of forms and thus covers a more diverse range than *Arabidopsis* (Gerats and Vandenbussche, 2005). While phenotypical studies regarding growth at sub-optimal temperatures exist (Warner, 2010), chilling responses of petunia at the level of molecular physiology have not been analyzed yet.

The chilling response of the plant phenotype reflects changes in the habitus like a reduced growth performance, which are the consequences of a disturbed plant metabolism and of the acclimation to the changed environment at transcriptional or post-transcriptional level. Usadel et al. (2008) showed for *Arabidopsis* rosettes that already small decreases of the ambient temperature cause changes in metabolism and expression of genes. Interestingly, this response to mild chilling revealed a remarkable similarity to the response to cold temperature of 4°C, but with a smaller extent of the reaction. The synthesis of cryoprotectants and stress metabolites was enhanced and leaf protein synthesis was up-regulated, accompanied by an increase of the protein content (Usadel et al., 2008). In the youngest mature source leaves of tomato, increased sugar, and starch levels were observed in response to a moderate reduction of temperature (day/night temperature: 16/14°C vs. 25/20°C; Venema et al., 1999). While especially at very low temperatures increases of osmolytes may protect cells from freezing (Mahajan and Tuteja, 2005), reduced export of carbohydrates might be responsible for reduced growth at chilling temperatures. Furthermore, particularly in thermophile plants, photosynthesis is considerably reduced after chilling due to impaired redox and circadian regulation (reviewed in Allen and Ort, 2001). This effect might underlie the impact of chilling on carbohydrate metabolism. Studies revealed that cold-induced inhibition of sucrose synthesis causes a phosphate-limitation of photosynthesis (Hurry et al., 2000). Cold acclimation of *Arabidopsis* leaves seems to be triggered by low phosphate, which induces changes in Calvin cycle enzymes like increased expression of Rubisco and sucrose biosynthesis enzymes (Hurry et al., 2000). Moreover, Usadel et al. (2008) reported a coordinated repression of some genes, responsible for starch and sucrose breakdown. Thus, transcript levels for vacuolar invertase as well as total invertase activity were reduced at low temperature.

Some phytohormones such as auxins, abscisic acid (ABA) and jasmonic acid (JA) are known to be involved in plant reactions to abiotic stress resulting from low temperatures. Auxin is a key regulatory phytohormone in plant growth and development, while indole-3-acetic acid (IAA) constitutes the most important physiologically active fraction. It plays an important role in plant reactions to environmental changes. Changed plant growth and development in response to cold may be linked to an altered intracellular homeostasis of auxins, which is regulated by local auxin gradients (reviewed in Rahman, 2013). Studies in *Arabidopsis* roots suggest that cold exposure affects auxin transport rather than auxin signaling. It is assumed that the intracellular trafficking of auxin eﬄux carriers is inhibited under cold stress (Shibasaki et al., 2009). Another important stress related phytohormone that plays a crucial role for chilling reaction is ABA. ABA synthesis seems to be essential for acclimation-induced chilling tolerance in maize seedlings (Anderson et al., 1994). Ntatsi et al. (2013) reported that tolerance of tomato to mild chilling stress was dependent on ABA biosynthesis, even though ABA accumulation itself seemed not to be essential for the acclimation mechanism. The biosynthesis and signaling of JA seems also to be important for maintaining normal physiological functions under cold stress (Du et al., 2013a; Hu et al., 2013).

Among the diverse mechanisms, providing plant plasticity toward a changing environment, the regulation of gene expression plays an important role in the genetic potential of a plant to cope with environmental stresses. In this context, the MYC-like transcription factor (TF) ICE1 from the bHLH family has a crucial control function in cold tolerance in *Arabidopsis.* ICE1 regulates the expression of several TFs that repress or activate further downstream cold-responsive genes (Chinnusamy et al., 2003; Lee et al., 2005). Other research disclosed an important role of the CBF family of TFs for adaptive changes of expression and metabolism in response to small decreases of temperature (Usadel et al., 2008). Recent studies proofed the important role of constitutive CBF expression for freezing tolerance also in petunia (Walworth et al., 2014). However, transcriptional profiling of petunia seedlings in response to cold at 2°C indicates that besides the CBF pathway diverse other regulatory pathways may exist that regulate the cold stress response (Li et al., 2015). Considering that the molecular and physiological response of petunia to water deficit follows both a severity- and time-dependent pattern (Kim et al., 2012), the transcriptional response to mild chilling may differ from the cold response to temperatures close or even below 0°C.

The molecular and physiological response of *P. hybrida* to mild chilling at the levels of carbohydrate metabolism, phytohormone homeostasis, and the transcriptome is unknown. In the present study, we monitored such responses in the chilling-sensitive cultivar ‘SweetSunshine Williams.’ This cultivar shows a strong growth depression during a period of 4 weeks under a temperature of 12°C when compared to 16°C, a temperature frequently used for greenhouse production of petunia in Germany. We followed the hypothesis that the growth depression is related to phase-specific changes in plant carbohydrate metabolism and/or phytohormone homeostasis. Therefore, we monitored carbohydrate levels and invertase activity by enzymatic assays and the phytohormones IAA, ABA, and JA by gas chromatography-tandem mass spectrometry (GC-MS/MS) in different aerial plant parts after differentiation of temperature. Furthermore, to obtain a comprehensive picture of the acclimation at gene expression level, we analyzed the transcriptome by use of a specific petunia microarray. This microarray provides 24,816 unique, non-redundant annotated sequences (Breuillin et al., 2010; Ahkami et al., 2014).

## Materials and Methods

### Plant Material and Sub-Optimal Temperature Treatment

All experiments were carried out with the chilling-sensitive *P. hybrida* cultivar ‘SweetSunshine Williams’ (‘Williams’), which shows a significant reduction in dry weight production, when exposed to sub-optimal temperature. As sub-optimal temperature, an average day temperature of 12°C was chosen and compared to an average day temperature of 16°C, which is commonly used in German greenhouse production, as control. With the cultivar ‘Williams,’ this decrease in temperature of 4 K causes a retarded growth without any visible damage due to cold stress. All plants, used for the experiments, were vegetatively propagated. Cuttings were rooted under greenhouse conditions. ED 73 petunia substrate was used for potting, but without slow-release fertilizers (Einheitserde Classic Tonsubstrat ED 73, +Fe, coarse, nutritive salt 1.0, without slow-release fertilizer (GEPAC LCD); Patzer GmbH & Co. KG, Sinntal, Germany). Salt and nutrient concentrations and pH of substrate were controlled during the experiments. Plants were fertilized with 0.15% Hakaphos spezial (16% N, 8% P_2_O_5_, 22% K_2_O, 3% MgO, and micronutrients; COMPO GmbH Münster, Germany) once a week.

Detailed growth performance analysis was conducted under greenhouse conditions (control temperature, day/night average: 16.8°C/15.2°C; sub-optimal temperature, day/night average: 13.1°C/11.1°C; 12 h photoperiod with ±130 μmol m^-2^ s^-1^ photo-synthetic photon flux density). All other experiments were conducted under climate chamber conditions. Therefore, immediately after potting, rooted cuttings were transferred to a climate chamber to acclimate to control temperatures (day/night: 17°C/15°C) and to develop a good root system. Day length was fixed to a 12 h photoperiod, (photo-synthetic photon flux density: during first week 100 μmol m^-2^ s^-1^, from second week onward 150 μmol m^-2^ s^-1^ (lighting: fluorescent tubes, FQ80W/865 HO Constant, Lumilux Cool Daylight, Osram, Germany), while the relative humidity was 65%. After 2 weeks of acclimation, half of the plants were moved to a climate chamber with identical conditions but under chilling exposure (day/night: 13°C/11°C). The 4-weeks period of cultivation focused on the vegetative growth of plants. To avoid interference with competing sinks, developing flower buds were continuously removed from the plants as soon as being visible during the course of the experiment.

### Growth Performance Analysis and Collection of Samples

For determination of growth, the main shoots of 12 plants per treatment were marked at the time point of 0 DoT (days after differentiation of temperature). As growth parameters, the number of newly developed shoots, the increase in length of the main shoot, and the number of newly developed leaves on the main shoot were evaluated weekly over a period of 28 days.

Samples were collected from three plant organs for analyses. As sink tissue, the apex of the main shoot was harvested, including adjacent small leaves (total length <2 cm). For sampling of source tissue, leaf disks (50–120 mg fresh weight for carbohydrates and 200–250 mg for phytohormones) were excised with a cork borer from the middle of each leaf half of young, but fully expanded leaves. For phytohormone and gene expression analyses, the uppermost internode (for microarray only at 21 DoT) was additionally collected. The samples were immediately transferred to liquid nitrogen and stored at -80°C until purification and analysis. The samples for carbohydrates, enzymes, and microarray were collected at 6 h, for phytohormone analyses at 8 h after begin of the photoperiod. Samples for carbohydrate, enzyme and phytohormone analyses were collected at 0, 1, 2, 3, 7, 14, 21, and 28 DoT, samples for the microarray analysis at 1, 3, 7, 21 DoT. Since metabolic and hormone data of 2 DoT gave no information in addition to those of 1 and 3 DoT, these were not included in the results.

### Analysis of Carbohydrates, Invertase Activity, and Phytohormones

Extraction, purification, and analysis of ABA, IAA, and JA by GC-MS/MS were performed as described by Ahkami et al. (2013). That protocol is a modification of the original protocol, described by Müller et al. (2002). To measure all three phytohormones, the 1 ml methanol, added to frozen samples for extraction, contained 10.3 pmol (^2^H)_2_-IAA, 10.3 pmol (^2^H)_6_-ABA and 27.8 pmol (^2^H)_6_-JA as internal standards. Gas chromatography and mass spectrometry settings for IAA, ABA, and JA were applied as described by Ahkami et al. (2013), Ntatsi et al. (2013), and Rasmussen et al. (2015). Ten biological replicates were analyzed per each treatment and date. Fructose, glucose, sucrose were extracted with 80°C aqueous ethanol. The sugars in the extract and starch in the extraction residue were analyzed in 96-well standard microplates using enzymatic assays as described by Hajirezaei et al. (2000) and Klopotek et al. (2010), using 12 biological replicates per treatment and date. Preparation and analysis of samples for invertase activities were conducted as described by Hajirezaei et al. (1993, 2000), using 10 biological replicates per treatment and date.

### Statistical Analyses

To execute statistical analyses, the STATISTICA software package was used^1^ [StatSoft, Inc. (2011); STATISTICA (data analysis software system), version 10]. The metabolite data were statistically analyzed by applying ANOVA in combination with student’s *t*-test, if normal distribution within the groups and variance homogeneity were met. Normal distribution was tested by Kolmogorov–Smirnov test and variance homogeneity by Levene’s test. If these assumptions were not fulfilled, a non-parametric test for comparing means (Mann–Whitney-*U* test or Kruskal–Wallis test) was applied and tested for significance (Ahkami et al., 2013). Significant differences at a level of *P* ≤ 0.05 were marked by asterisks in the respective figures.

### Microarray Hybridization and Statistical Analysis

In order to identify genes with altered expressions in response to sub-optimal temperatures, a petunia-specific microarray was hybridized, which carries 24,816 unigene annotated sequences (Ahkami et al., 2014) and was first described by Breuillin et al. (2010). RNA was extracted from three replicates by the QIAGEN kit (Qiagen, Hilden, Germany). Each replicate was a pooled sample of four individual plants. For extraction, RNA was treated with DNase, following the Qiagen protocol. A minimum of 500 ng total RNA per sample was required for array hybridization, performed by Oaklabs (OakLabs GmbH, Hennigsdorf, Germany). Normalization of the whole set of data was conducted by Oak Labs using the Quantil-normalization according to Bolstad et al. (2003). For analysis of each time point (1, 3, 7, and 21 DoT), expression values derived from samples grown at sub-optimal temperature (12°C) and at control temperature (16°C) were compared. *M*-values (Log_2_ of ratios) were used to demonstrate the intensity of up- or down-regulation of genes under the exposure to chilling. Log_2_ >1 was defined as up-regulated, Log_2_ <-1 was defined as down-regulated. For identification of statistically significant up- or down-regulated genes, a Rank Product online-analysis^2^ was carried out (Breitling et al., 2004; Laing and Smith, 2010). Hereby, a pfp (probability of false prediction) value threshold <0.15 was applied to identify statistically significant differentially expressed genes. Expression graphs were created with Genesis, software version 1.7.6. (Sturn et al., 2002). Information regarding TFs in *Arabidopsis thaliana* was provided by the Stress Responsive Transcriptions Factor Database (STIFDB^3^; Naika et al., 2013).

## Results

### Growth Response to Mild Chilling

The chilling-sensitive *P. hybrida* cultivar ‘Williams’ reacted to the reduction of mean ambient temperature by 4 K with a strong decrease in growth and a retarded development. After 28 days, shoot dry and fresh weights were reduced by 37 and 43%, respectively (Supplementary Figure S1). This was associated with reductions of the elongation of the main shoot by 60% (Supplementary Figure S2A) and the number of newly developed leaves by 40% (Supplementary Figure S2C). However, the branching (Supplementary Figure S2B), i.e., the development of new shoots, was not affected by the chilling temperature.

### Response of Carbohydrate Levels and Invertase Activities to Mild Chilling

**FIGURE 1:**
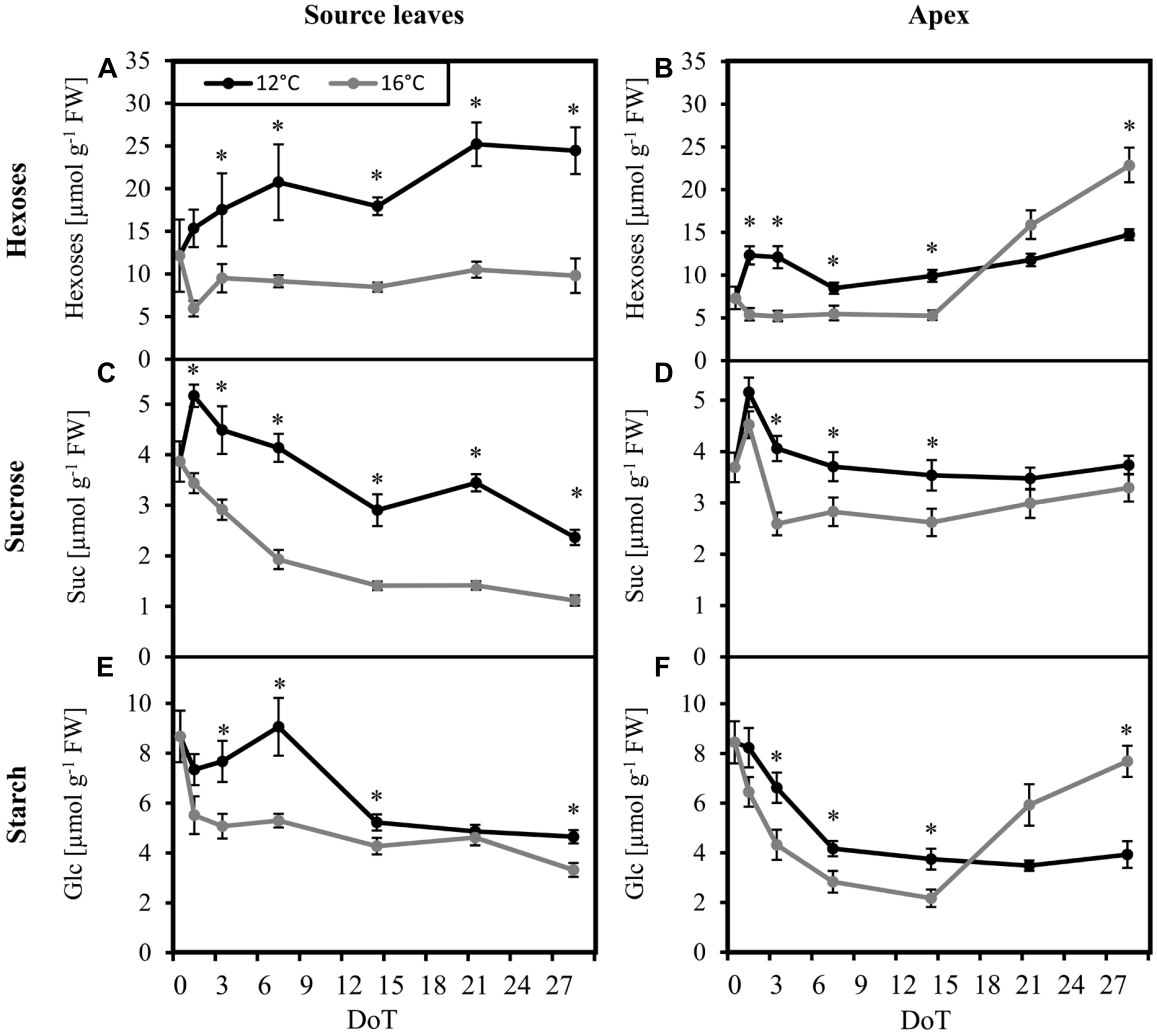
**Carbohydrate levels.** Impact of sub-optimal temperature (black: 12°C, gray: 16°C) on carbohydrate levels. **(A)** source leaves, hexoses; **(B)** apex, hexoses; **(C)** source leaves, sucrose; **(D)** apex, sucrose; **(E)** source leaves, starch [specified in units of glucose (Glc)]; **(F)** apex, starch (specified in units of Glc). (DoT = days after differentiation of temperature; *n* = 12; data are means ± SE; asterisks indicate significant differences between temperature treatments for a given sampling time, *P* ≤ 0.05).

Considering the crucial role of carbohydrates as protective molecules for plant survival at very low and freezing temperatures, we analyzed the concentrations of the most important sugars fructose (Fru), glucose (Glc), and sucrose (Suc) as well as starch in source leaves and in the shoot apex as important utilization sink. Samples were collected at midday, 6 h after the start of the photoperiod, at the expected climax of sugar concentrations (Nägele et al., 2010). A strong response of the carbohydrate levels to the chilling treatment was observed. However, the dynamic was different in source and sink tissues. During the first days of chilling exposure, concentrations of hexoses (Fru + Glc; **Figure 1A**), Suc (**Figure 1C**) and starch (**Figure 1E**) in source leaves increased significantly compared to the respective values of control plants. While hexose and Suc concentrations remained on significantly higher levels throughout the experiment, starch concentrations dropped down to values only slightly higher than in the control source leaves at 14–28 DoT with no significant difference at 21 DoT. In the shoot apex, especially hexoses, and starch displayed a chilling response that was composed of three phases, a fast, a midterm and a long-term response. At first, concentrations of hexoses (**Figure 1B**), Suc (**Figure 1D**), and starch (**Figure 1F**) increased under chilling stress similar to the response in the source tissue. However, the increase in the apex was smaller for all three fractions when compared to the leaves. After one week at sub-optimal temperature (midterm reaction), the concentrations of Suc and starch in the apex of chilled plants reached a steady state, which did not change thereafter, while hexose levels showed a slight increase. At control temperature, however, concentrations of hexoses and starch displayed a sharp increase during week three and four, resulting in significant higher concentrations at 28 DoT. This led to significant lower hexose and starch concentrations in the apex after prolonged exposure to the chilling temperatures when compared to the control plants. Such relations were not observed in the source tissue.

**FIGURE 2:**
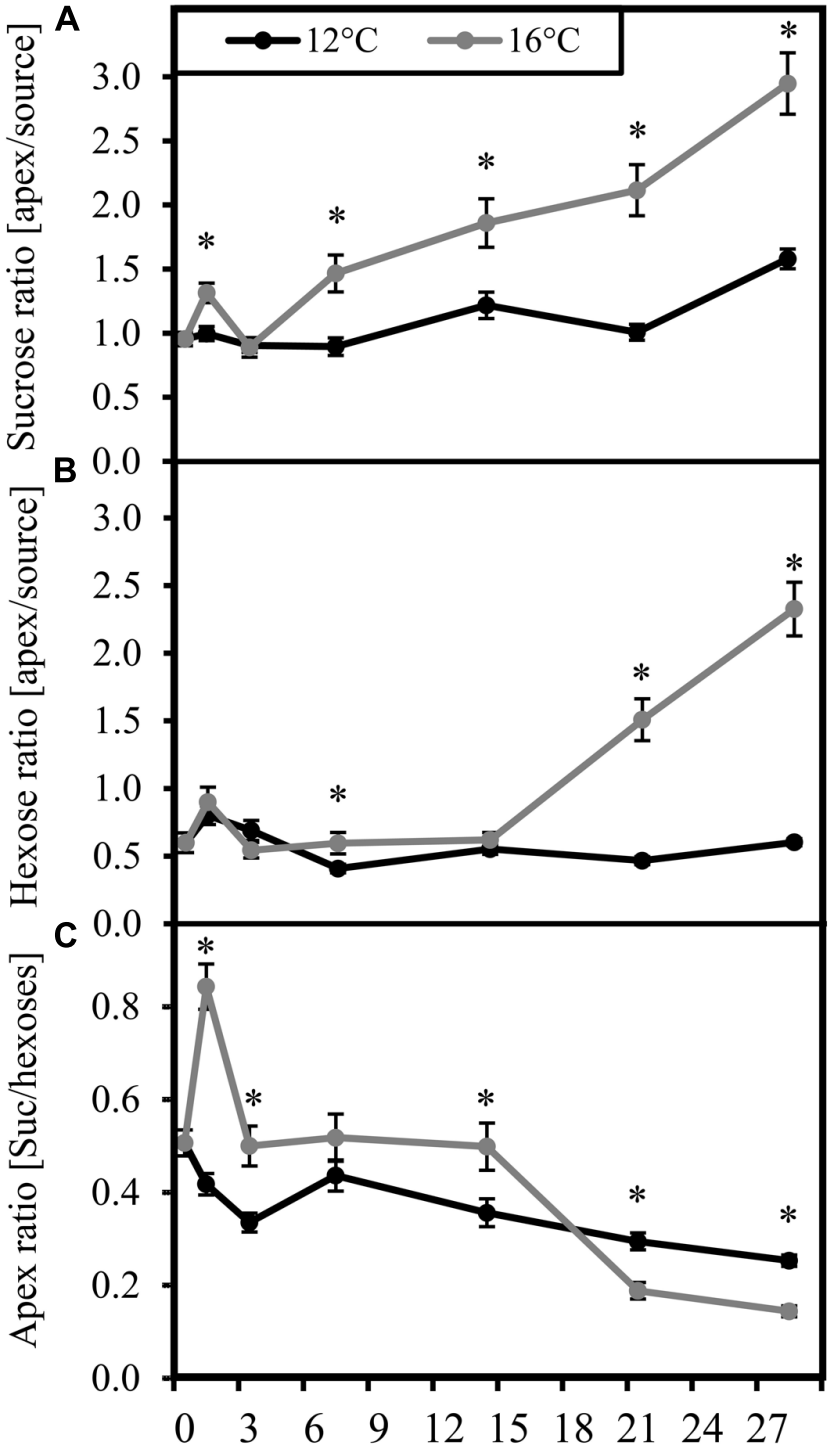
**Carbohydrate ratios.** Ratios (black: 12°C, gray: 16°C) of carbohydrate concentrations. **(A)** apex/source ratio of sucrose; **(B)** apex/source ratio of hexoses; **(C)** sucrose/hexose ratio in the apex. (DoT = days after differentiation of temperature; *n* = 12; data are means ± SE, asterisks indicate significant differences between temperature treatments for a given sampling time, *P* ≤ 0.05).

While carbohydrate concentrations in the tissues at certain time points are snap-shots of current carbohydrate availability, carbohydrate ratios between source and sink and among each other may indicate bottlenecks of partitioning between carbohydrate fractions and between tissues at the two different temperatures. It becomes apparent from **Figure 2**, that cultivation at the sub-optimal temperature strongly reduced the ratio of carbohydrate levels between sink and source tissue when compared to the control plants. This difference started with sucrose (**Figure 2A**) at 7 DoT and was followed by the hexoses (**Figure 2B**) at 14 DoT. Exposure to sub-optimal temperature caused a transient reduction of the sucrose/hexose ratio in the apex until 14 DoT, which, however, from 21 DoT onward was followed by a reversed situation with higher sucrose/hexose ratios in the chilled plants (**Figure 2C**) To sum up, the carbohydrate levels and ratios indicate that the chilling treated plants accumulated sugars in the source leaves at the expense of carbohydrate transport to the shoot apex as utilization sink. In addition, from 21 DoT onward, Suc utilization in the shoot apex seemed to be inhibited as indicated by the increased Suc/hexose ratio in the apex.

**FIGURE 3:**
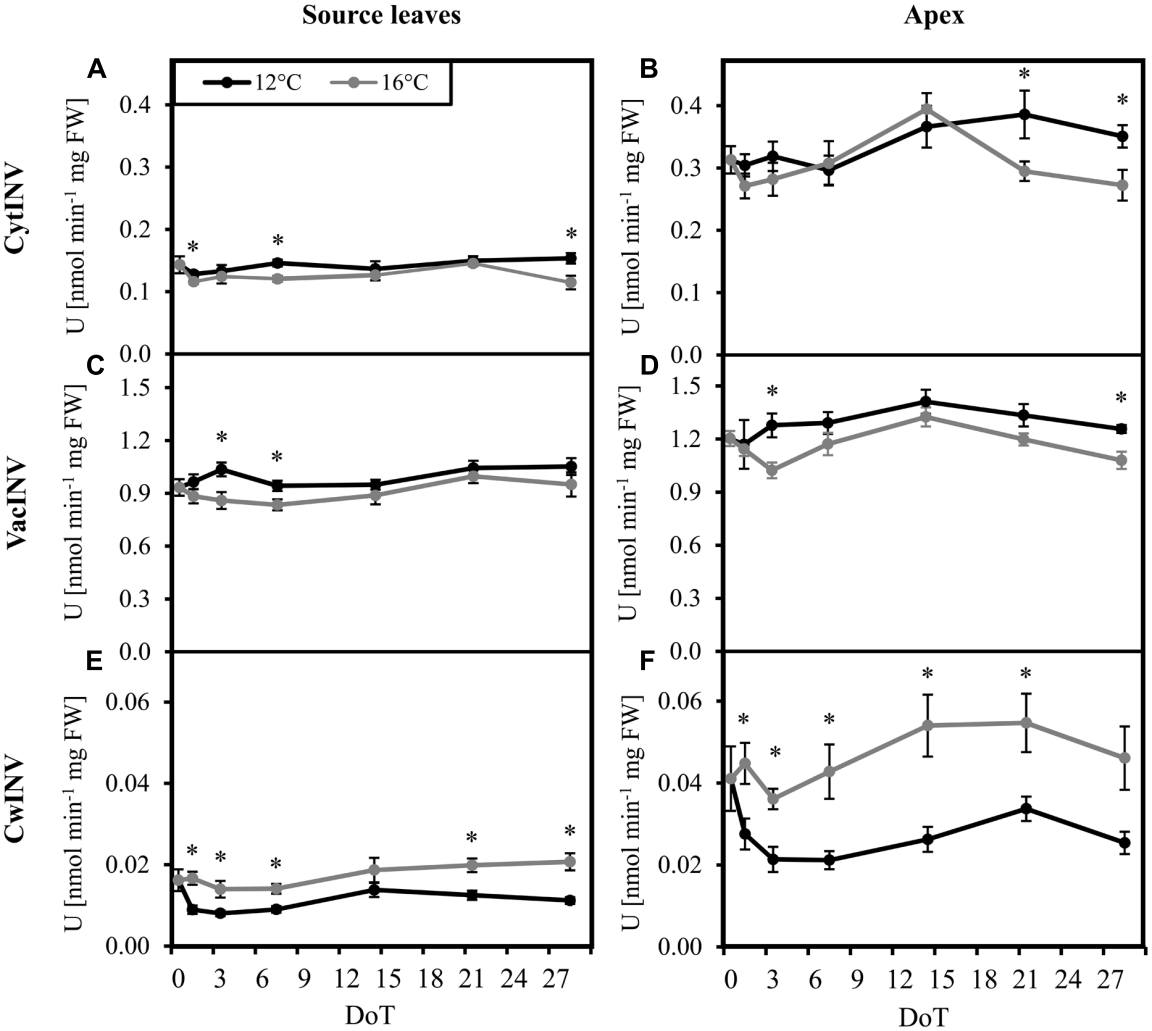
**Invertase activities.** Impact of sub-optimal temperature (black: 12°C, gray: 16°C) on invertase activities. **(A)** source leaves, cytoplasmic invertase (cytINV); **(B)** apex, cytoplasmic invertase; **(C)** source leaves, vacuolar invertase (vacINV); **(D)** apex, vacuolar invertase; **(E)** source leaves, cell wall invertase (cwINV); **(F)** apex, cell wall invertase. (DoT = days after differentiation of temperature; *n* = 10; data are means ± SE, asterisks indicate significant differences between temperature treatments for a given sampling time, *P* ≤ 0.05).

Considering the changed carbohydrate levels and particularly the changed Suc/hexose equilibrium, we investigated the activities of cell wall bound (cwInv), cytoplasmic (cytInv), and vacuolar (vacInv) forms of invertase. In both, source and sink tissues, activities of cytInv (**Figures 3A,B**) and vacInv (**Figures 3C,D**) showed a slight, but mostly not significant increase of activities under chilling stress. In contrast, the activity of cwInv was significantly reduced under chilling stress, with a stronger absolute decrease of activity in the apex (**Figures 3E,F**).

### Response of Phytohormone Levels to Mild Chilling

**FIGURE 4:**
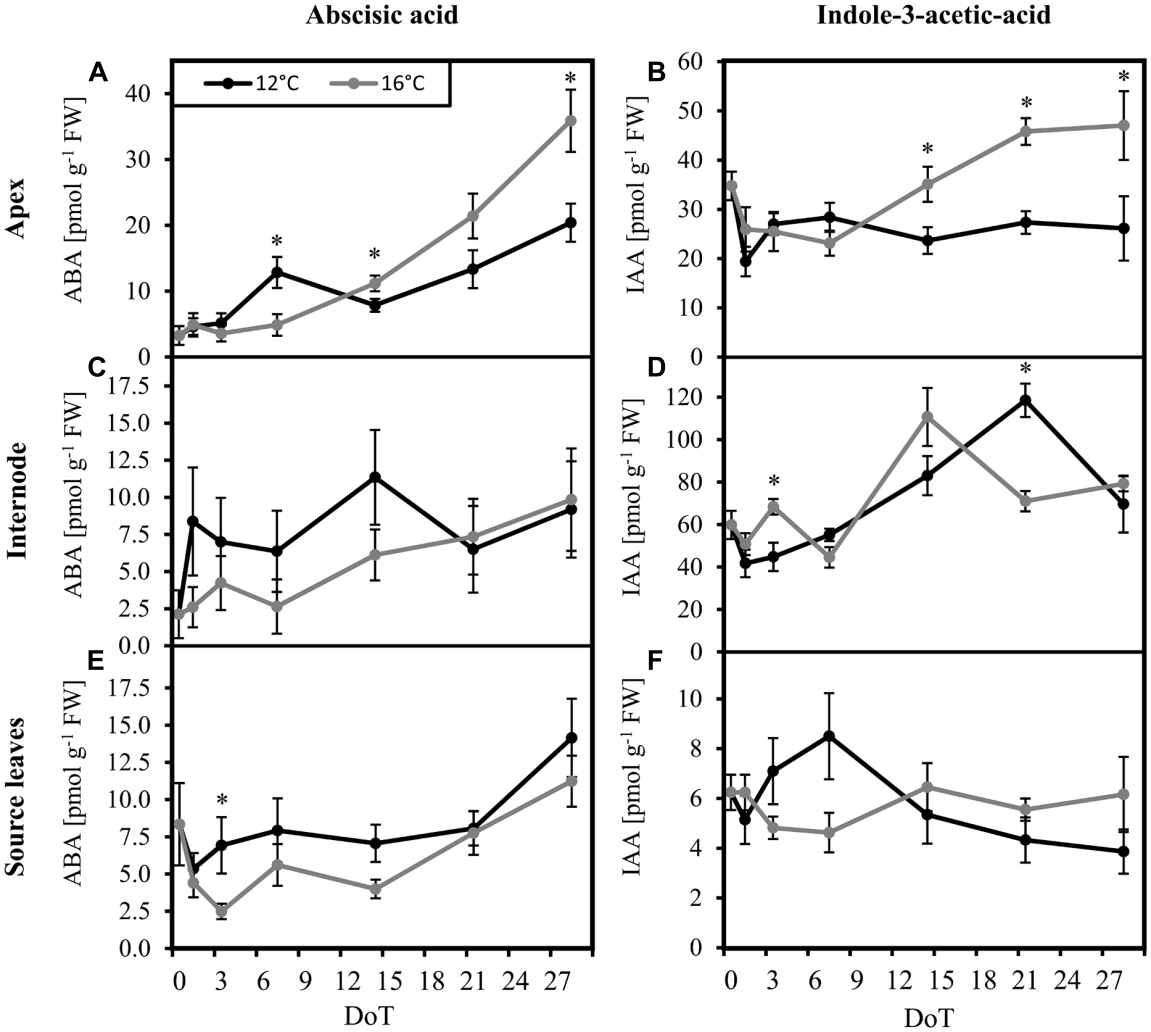
**Abscisic acid and indole-3-acetic-acid levels.** Impact of sub-optimal temperature (black: 12°C, gray: 16°C) on phytohormone levels. **(A)** apex, abscisic acid (ABA); **(B)** apex, indole-3-acetic-acid (IAA); **(C)** internode, ABA; **(D)** internode, IAA; **(E)** source leaves, ABA; **(F)** source leaves, IAA. (DoT = days after differentiation of temperature; *n* = 10; data are means ± SE; asterisks indicate significant differences between temperature treatments for a given sampling time, *P* ≤ 0.05).

In order to unravel if the homeostasis of ABA, IAA, and JA in petunia is altered by exposure to sub-optimal temperature, concentrations of these phytohormones were monitored. Since the stem provides an important transport unit for phytohormones and, furthermore, IAA may control stem-elongation, the uppermost internode was analyzed in addition to the source leaves and the shoot apex. The mild chilling treatment had no measureable impact on levels of JA. For all three tissues, JA concentrations were very low. Actually, most values were below the limits of quantification of 1.5 pmol per injection (Rasmussen et al., 2015) for both treatments (data not shown). Therefore, an evaluation of JA homeostasis was not possible. However, the concentrations of ABA and IAA in the tissues changed in response to the exposure to sub-optimal temperature. When compared with the control plants, at 3 DoT and 7 DoT the chilled plants contained higher ABA concentrations in the source leaves and in the apex, respectively. ABA concentrations in the apical sink tissue accumulated during the last 2 weeks of the experiment. However, at 28 DoT the ABA concentration in the apex was significantly lower in chilled plants than in control plants (**Figure 4A**). During this period, ABA concentrations neither in the internodes (**Figure 4C**) nor in the source tissue (**Figure 4E**) were affected by temperature. IAA concentrations in the apex were not significant different during the first week (**Figure 4B**). However, during week two and three, concentrations in control plants increased and seemed to reach a steady plateau in week four, whereas concentrations in the chilled plants remained on a significantly lower level, comparable to that at 7 DoT. IAA concentrations in the internodes (**Figure 4D**) were on a generally higher level than in source leaves and increased under chilling treatment continuously from 1 DoT to 21 DoT, without showing the fluctuations, that were observed at 16°C. IAA concentrations in the source tissue (**Figure 4F**) did not vary significantly between 12 and 16°C.

### Response of Gene Expression to Mild Chilling

**FIGURE 5:**
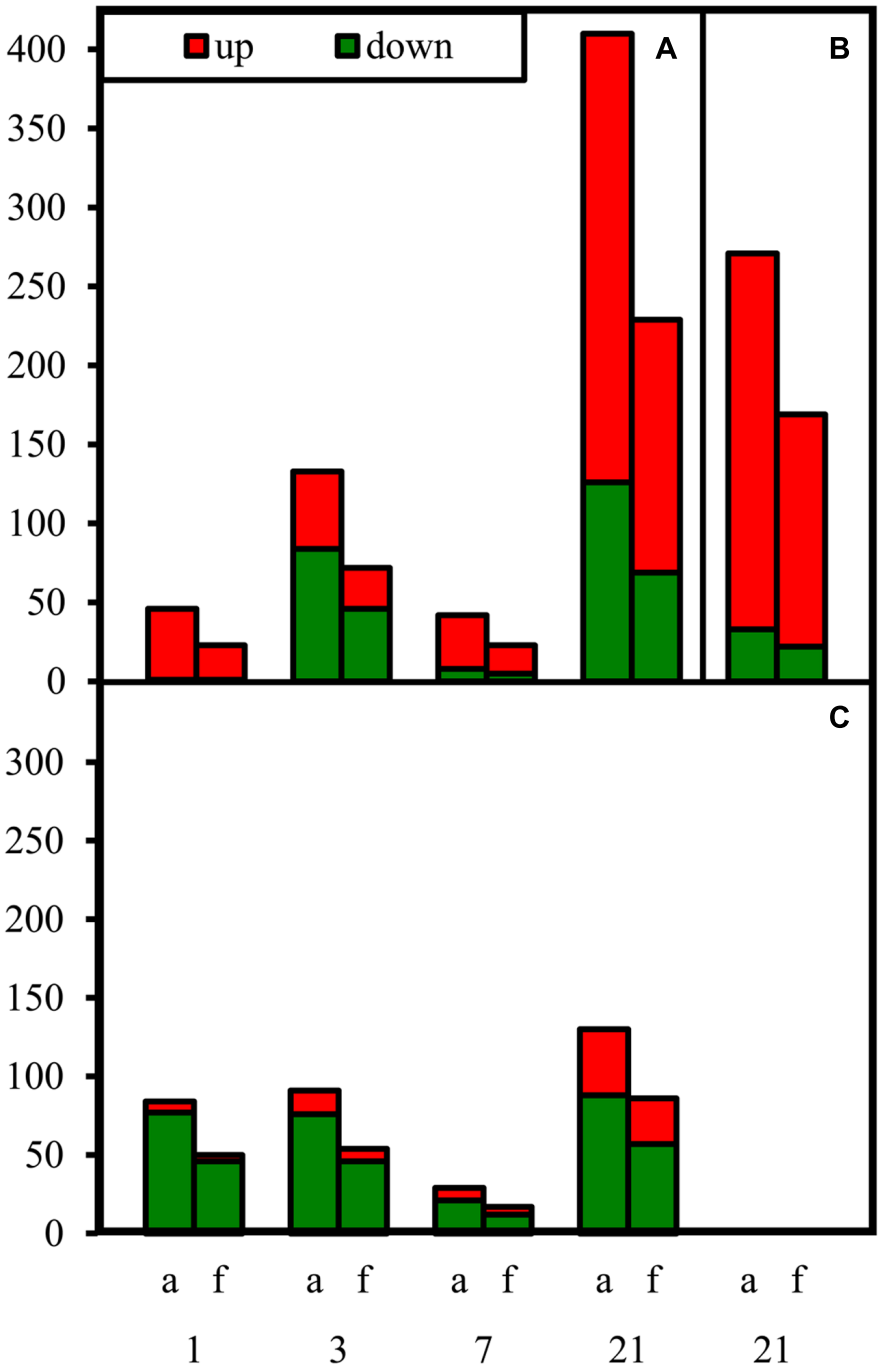
**Numbers of differentially expressed genes under chilling stress.** Numbers of down- and up-regulated genes (down: chilling < control; up: chilling > control) at different dates after differentiation of temperatures. **(A)** source leaves at 1, 3, 7, 21 DoT; **(B)** internode at 21 DoT; **(C)** apex at 1, 3, 7, 21 DoT. (a, all sequences, f, genes annotated with a known protein function; RankProduct calculated pfp-values <0.15; upregulated: Log_2_ >1; down-regulated: Log_2_ <-1).

Samples were collected on four dates (1, 3, 7, 21 DoT), to examine fast, midterm and long-term reactions of gene expression to mild chilling temperatures. Even the 4 K reduction of temperature caused significant changes in gene expression. Most M values (log_2_ of fold change ratios) for differentially expressed genes were between -1 and -3 for down-regulated or between 1 and 3 for up-regulated putative genes, respectively. However, a few genes showed even *M*-values below -3 or above 3. **Figure 5** shows the numbers of significantly differentially expressed genes for the different dates and plant tissues. The response patterns were similar for (a) all significantly differentially expressed genes and (f) those with an annotated function. Numbers of differentially expressed genes disclose three phases of stress-reaction, an early phase between 1 and 3 DoT, a midterm phase at 7 DoT, and a late phase at 21 DoT. Furthermore, the chilling response was in general different between the two tissues with more down-regulated genes in the apex (**Figure 5C**) and more up-regulated genes in the source leaf (**Figure 5A**). Interestingly, the response pattern in the internode, which was analyzed only at 21 DoT, was similar to that of the source leaves (**Figure 5B**). The following analysis focuses on the group of genes with annotated functions only.

The transcription of genes in the source tissue (**Figure 5A**) as well as in the shoot apex (**Figure 5C**) responded to chilling within the first day. This fast response was mainly marked by up-regulation in the source leaves (up/down: 22/1) but by down-regulation in the apex (up/down: 4/46). The apex type of response did not change between 1 and 3 DoT (8/46). In source leaves, however, a strong increase in the number of down-regulated genes was observed at 3 DoT reaching the same number as in the apex (46). During the midterm phase (7 DoT), numbers of differentially expressed genes were sharply reduced in both tissues. The decrease in numbers was mainly due to less down-regulated genes in the apex (12) and in the source leaf (5). However, the midterm reaction was followed by a phase of strong regulation of gene expression, so that highest numbers of up- and down-regulated genes were reached at 21 DoT. Then, most up-regulated genes were found in the source leaf (160) compared to down-regulated genes (69) and to the apex (29), while a high number of up-regulated genes (147) was also detected in the internode (**Figure 5B**).

**FIGURE 6:**
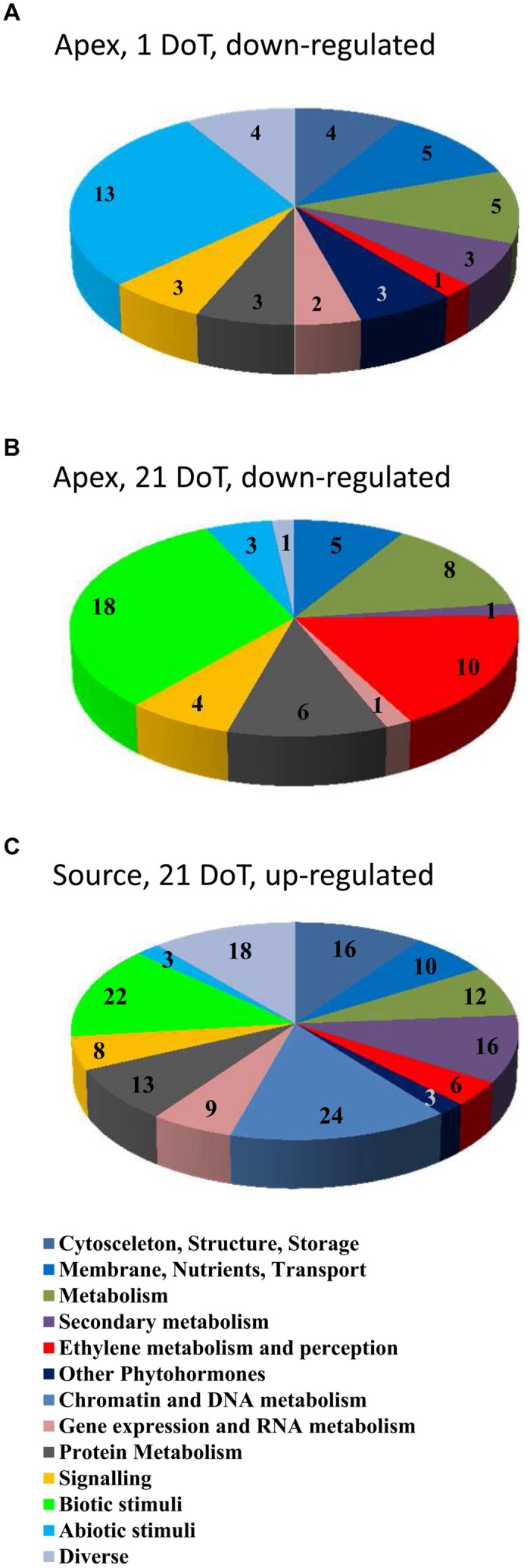
**Chilling response of functional groups.** Numbers of regulated genes of functional groups (groups see Supplementary Table S1D). Down-regulated in the apex at 1 **(A)** and 21 DoT **(B)** and up-regulated in the source at 21 DoT **(C)**. (Up-regulated: log_2_ > 1; down-regulated: log_2_ <-1; pfp-value <0.15).

The differentially regulated genes belonged to various functional groups, whose response to mild chilling varied regarding date and plant organ. Numbers of regulated genes in the different groups are illustrated in **Figure 6**, distinguishing between down-regulated genes in the apex at 1 DoT (A) and 21 DoT (B) and up-regulated genes in the source at 21 DoT (C). The groups are identical to or merged of functional classes as indicated in Supplementary Table S1D. At 1 DoT, the functional group “abiotic stimuli” was down-regulated in the apex (**Figure 6A**) without showing any up-regulation in the same tissue or any down-regulation in the source (Supplementary Table S1). The organ-specific response of individual functional groups to the mild chilling became also apparent after longer exposure to the sub-optimal temperatures. At 21 DoT, the functional groups “biotic stimuli” and “ethylene metabolism and perception” were strongly down-regulated in the apex (**Figure 6B**) but not in the source tissue (Supplementary Table S1). However, simultaneously a similar quantity of genes of both groups and the group “chromatin and DNA metabolism” were up-regulated in the source tissue (**Figure 6C**).

**FIGURE 7:**
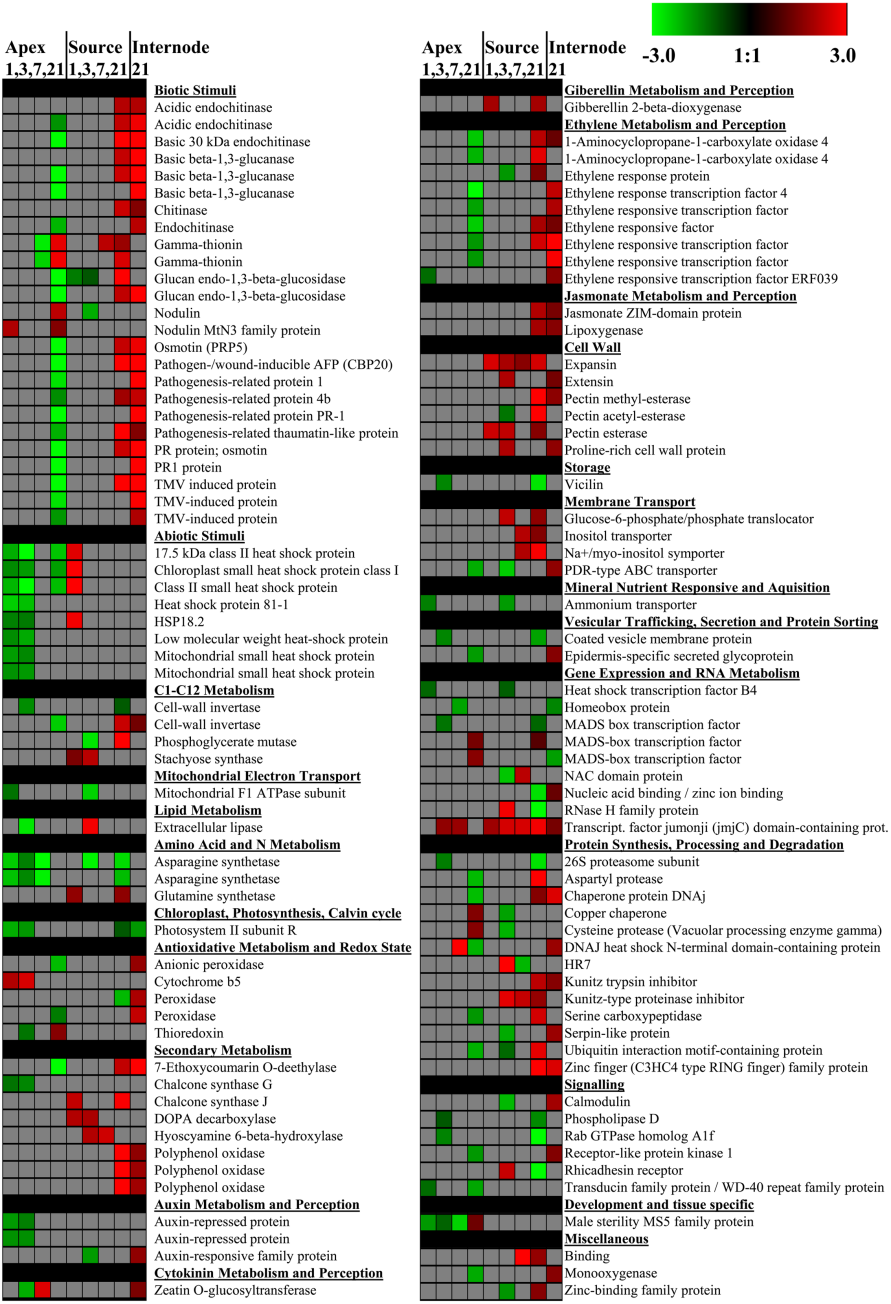
**Individual differentially expressed genes.** Impact of sub-optimal temperature on expression of individual genes with annotated putative function. Sub-optimal temperature-induced changes are shown for the apex (1, 3, 7, 21 DoT), source leaves (1, 3, 7, 21 DoT), and the internode (21 DoT). The figure focuses on genes, which were differentially expressed at least at two dates or in two organs, respectively. Colored squares represent Log_2_ expressions values for individual genes: red color represents up-regulated genes, green color represents down-regulated genes. Intensity of color indicates extend of change in expression (pfp-value < 0.15).

**Figure 7** considers individual genes and focuses on those, which were differentially expressed at least at two dates, respectively, in two tissues. Supplementary Table S1 shows the full list of all significant differentially regulated genes including their SEQ_IDs. With respect to a particular tissue, most of those genes, which showed regulation at least at two dates, responded in the same direction at different time points. However, only two genes showed consistent expression patterns over all time points analyzed and this was restricted to the source leaves (details discussed below). Comparing the two tissues, contrasting patterns became obvious between the source leaves and the apex. Several individual genes in the functional groups “Abiotic stimuli” and “Biotic stimuli” were down-regulated in the apex, but simultaneously up-regulated in the source leaf and partially in the internode. This applies to some genes of the category “Abiotic Stimuli’ at 1 DoT and particularly to genes related to the group “Biotic stimuli” at 21 DoT. Among these were especially genes, which are normally induced by pathogen-induced plant defense responses. Overlaps of genes with the same direction of regulation between source leaf and apex were low in general. However, 49 up-regulated genes overlapped between the source leaves and the internode on 21 DoT, while 28 of these overlapping genes had an annotated protein function.

Also at the level of individual genes, three phases of chilling response became apparent (**Figure 7**). An active early phase during the first 3 days of chilling was marked by the phase-specific and apex-restricted down-regulation of five genes of the category ‘Abiotic Stimuli’ coding for heat shock proteins and of four genes encoding one Photosystem II subunit, one chalcone synthase G and two auxin-repressed proteins. The subsequent midterm phase after 1 week (7 DoT) with only few genes regulated was followed by a hyper-active gene regulation after 3 weeks (21 DoT). At this time, seven individual genes of the functional group “Ethylene metabolism and perception” were down-regulated in the apex but at the same time up-regulated in the source leaf and/or in the internode (**Figure 7**). These genes were two genes coding for 1-aminocyclopropane-1carboxylate oxidase 4 (SEQ_ID in Supplementary Table S1B: cn2105) and different types of ethylene TFs. Within the category, “Auxin metabolism and perception,” one gene coding for a particular auxin-responsive family protein was down-regulated in the late phase in the internode (**Figure 7**). However, no significantly down- or up-regulated genes were found in the functional group of “Abscisic acid metabolism and perception.” Looking at “Jasmonate metabolism and perception,” two differentially expressed genes were found, both up-regulated in source leaves and the internode at 21 DoT (**Figure 7**). One of them was a gene coding the jasmonate ZIM-domain protein (JAZ, SEQ_ID cn5086), a negative regulator in jasmonate-induced gene expression (Ishiga et al., 2013).

Genes related to different stages of carbohydrate metabolism were regulated under chilling stress. For example, one gene coding for the mitochondrial F1 ATPase subunit, a PS light reaction ATP synthase (GO_drs21P0003D18_R_ab1), was down-regulated in the apex at 1 DoT and in the source at 3 DoT. Three genes coding for AAA-type ATPase were up-regulated at 21 DoT, two in the source leaves (DY395838_1, GO_drs21P0002F21_R_ab1) and one (GO_drs12P0011F24_F_ab1) in the internode. One gene coding for photosystem II subunit R (cn675) was down-regulated in the apex within the early phase (1 and 3 DoT) but in the source and in the internode at 21 DoT. At the same date, down-regulation of four genes coding for RuBisCO activase (cn2051, cn2052, cn2053, cn3274), a type of chaperone that is essential to promote and maintain the catalytic activity of RuBisCO (reviewed in Portis, 2003) further indicated trancriptional inhibition of photosynthesis particularly in the internode. Looking at invertases, three of four cwINV genes were differentially regulated (**Figure 7**; Supplementary Table S1B). In the source leaves, one gene (cn8044) was up-regulated by chilling at 1 DoT and 21 DoT, whereas one other gene (GO_drpoolB-CL9414Contig1) was down-regulated at 21 DoT. However, in the apex chilling repressed the same gene at 3 DoT and two other genes (cn5583, cn8044) at 21 DoT. One gene coding for an invertase inhibitor (cn8301) was down-regulated in the source at 3 DoT whereas one other (GO_drpoolB-CL5724Contig1) was up-regulated in the internode at 21 DoT.

**FIGURE 8:**
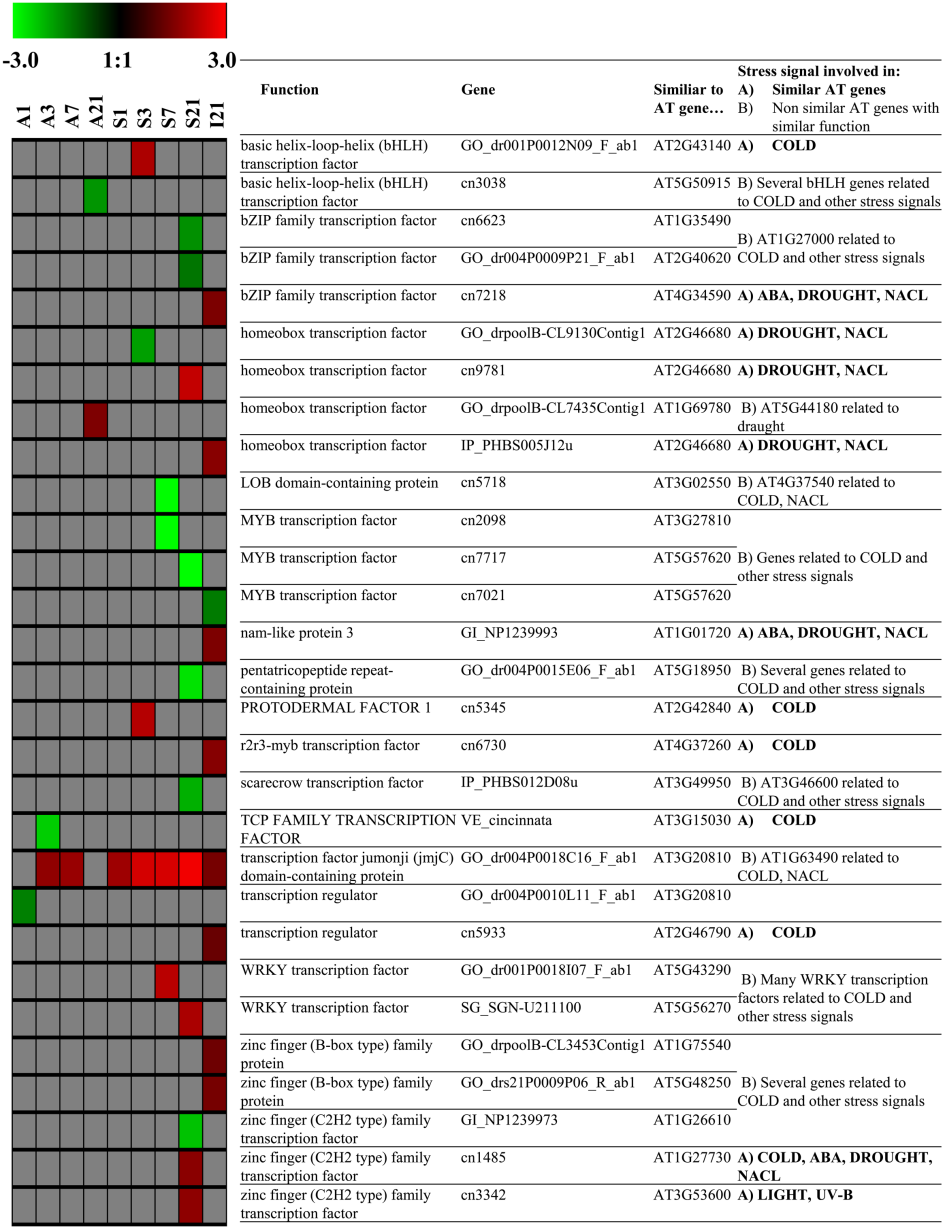
**Transcription Factors.** Impact of sub-optimal temperature on expression patterns of transcription factors (TFs) related to TFs that are involved in stress signaling in *Arabidopsis thaliana*. Group **(A)** genes similar to *Arabidopsis* genes coding for TFs related to stress, group **(B)** other genes coding for TFs that belong to TF families in *Arabidopsis* that are related to stress (derived from STIFDB V2.0). Colored squares represent Log_2_ expressions values for individual genes: red color represents up-regulated genes, green color represents down-regulated genes. Intensity of color indicates extend of change in expression (pfp-value < 0.15).

One gene coding for expansin (GO_drpoolB-CL8367Contig1), which is required for leaf growth, was constitutively up-regulated in the source at all dates (1/3/7/21 DoT, **Figure 7**). Also in the source, 17 genes coding for histones H2, H3 and H4 (cn1033, cn1034, cn1037, cn1359, cn1360, cn1361, cn3286, cn4604, cn5095, cn5315, cn5413, cn5414, cn5953, cn8099, cn9045, cn9421, EB174394_1) were up-regulated only at 21 DoT. In addition, two genes coding for chromomethylase (cn9108, GO_drs12P0025N23_F_ab1), responsible for DNA-methylation (Du et al., 2013b), were up-regulated at 21 DoT. Interestingly, in the apex genes coding for histones did not respond to the chilling stress. **Figure 8** shows differentially expressed TFs that (A) are similar to stress related TFs in *Arabidopsis thaliana* or (B) belong at least to gene-families that are known to include TFs that are involved in stress reactions. A noticeable higher number of differentially expressed TFs, involved in stress signaling, was found in the source tissue and the internode, compared to the apex. TFs showed the strongest reaction to chilling stress at 21 DoT, correlating with the overall higher numbers of differentially expressed genes in the source at 21 DoT, compared to earlier dates and compared to the apex. Differentially regulated genes were found within the TF families of basic helix-loop-helix (bHLH), bZIP family, homeobox, MYB, WRKY, zinc finger family, and others. However, the most continuously affected gene with regard to both, dates and tissues, was a gene coding for the TF Jumonji (jmjC) domain containing protein (GO_dr004P0018C16_F_ab1). It was consistently up-regulated in the source at all dates, in the apex at 3/7 DoT, and in the internode at 21 DoT (**Figure 8**).

## Discussion

Exposure of the chilling-sensitive *P. hybrida* cultivar ‘Williams’ to sub-optimal temperature led to a clear growth depression. The strong reduction of shoot elongation and leaf number contrasting to the absent response of shoot number (Supplementary Figure S2) indicates that sub-optimal temperature impaired growth of individual shoots whereas branching rate by outgrowth of new lateral shoots seems to be less sensitive to moderate chilling. This stays in accordance with similar findings on another *P. hybrida* cultivar reported by Ilias and Rajapakse (2012).

Our findings indicate that the observed growth depression is the outcome of a functional disturbance of the whole plant resulting from a highly dynamic and complex molecular physiological stress response. This is mirrored by changes in carbohydrate metabolism, phytohormone homeostasis, and gene expression patterns in three aerial parts of the plant, which constitute important functional units for plant growth.

### Mild Chilling Stress Causes Sugar Accumulation in the Source Leaves at the Long-Term Expense of Carbohydrate Supply to the Apex

Carbohydrate metabolism provides the backbone of plant growth, which is dependent on the availability of carbon sources in the plant and on the transport from source tissues to growing sinks. Furthermore, growth is dependent on carbohydrate utilization at the site of growth. Up to now, only few studies have investigated the response of carbohydrate metabolism to mild chilling temperatures and those studies did not consider different functional units of the plant. Venema et al. (1999) reported increased concentrations of water-soluble carbohydrates and starch accumulation in the youngest mature source leaves of tomato in response to mild chilling temperatures (day/night temperature, control: 20/25°C; chilling: 16/14°C). Nägele et al. (2011) investigated the cold-response (day/night temperature, control: 22/16°C; chilling: 4/4°C) of *Arabidopsis* for a period of 3 days and proposed three consecutive stages of cold acclimation of rosette leaves: an instant dislocation of homeostasis, followed by a restoration of the carbohydrate metabolism and finally a stabilized new homeostasis of carbohydrate metabolism.

To cover the dynamic of both sides of the source-sink network of aerial parts of petunia plants, we monitored carbohydrate levels in the uppermost fully developed leaves as carbohydrate source tissue and in the shoot apex as important utilization sink over a period of 28 days. Even though the microarray data revealed a disturbance of the photosynthetic machinery at transcriptional level, the metabolic data does not reflect a carbohydrate shortage at the source side. By contrast, ‘Williams’ reacted to exposure to sub-optimal temperatures with a sudden strong increase of hexoses and sucrose already within the first day of treatment, followed by an accumulation of starch from the second day on. While the accumulation of carbohydrates in source leaves was maintained over the whole chilling period of 4 weeks, the increase of carbohydrates in the apex during the first days was followed by intermediate approximation to concentrations of the control between 7 and 14 DoT, and from 21 DoT onward, turned into a reversed situation with lower hexose and starch levels in chilled plants when compared to the controls (**Figure 1**). The carbohydrate ratios between apex and source leaves (**Figure 2**) further highlight the long-term source-sink decoupling under mild chilling stress. While a development-related increase of the apex/source sugar ratios over the time in the control plants indicates an increasing supply of the apex with carbohydrates during the course of plant growth, this change was almost absent under the exposure to chilling temperatures.

Soluble sugars are well known to increase in plant cells at low temperatures and to act there as cryoprotectants that shelter plant cells from freezing (reviewed in Mahajan and Tuteja, 2005). The present carbohydrate data indicates, that the acclimation of the chilling-sensitive petunia cultivar even to the low stress level follows a “defense priority” to protect the source leaves, which have an important maintenance function for the plant. However, sugar accumulation in source tissues competes with carbohydrate export to the sinks. Obviously, under prolonged chilling stress translocation of carbohydrates to the shoot apex and/or their utilization in the apex were reduced and this probably contributed to the growth depression. The observed source-sink decoupling may involve inhibition of carbohydrate export from leaves, of the transport route between the source and sink tissue and also of the sink activity itself, which further drives carbohydrate influx. In this context, the higher sucrose/hexose ratio in the apex of chilled plants from 21 DoT onward indicates a changed sugar equilibrium, which may result from impaired enzymatic sucrose cleavage at the sink side.

### Reduction of Activity and Expression of Cell Wall Invertases Particularly in the Apex, but also Other, Post-Transcriptional Processes Seem to be Involved

Since invertases, which are responsible for the turnover of Suc to Fru and Glc and thereby contribute to sink activity (Ahkami et al., 2013), can be affected by low temperature (Usadel et al., 2008), we investigated the activities of cell wall bound (cwInv), cytoplasmic (cytInv), and vacuolar (vacInv) forms of this enzyme. Activities were measured under standard conditions at optimized temperature of 37°C (Ahkami et al., 2013) providing high turnover rates to detect acclimation of invertases to chilling stress. Additional tests at different measuring temperatures (data not shown) showed that the turnover rates per unit time at 16°C, respectively, 12°C were too low for an accurate comparison of enzyme activities at the different current temperatures. Taking into account the general temperature dependency of enzyme activity, the slightly increased levels of vacINV and cytINV activity (**Figures 3A–D**) might even indicate a lower *in vivo* activity at the colder temperatures. Nevertheless, it is an indication for a slight up-regulation of the two invertases particularly in the apex, which might indicate a compensatory response to the above-mentioned insufficient sucrose cleavage. By contrast, the significantly reduced activity of cwINV after exposure of plants to chilling temperatures (**Figures 3E,F**) reflects an adaptive down-regulation of enzyme activity in response to chilling and is further a strong indication for a chilling-induced reduction of *in vivo* cwINV activity at the lower temperatures, especially in the apex. Considering that correspondingly genes coding for cwINV were found to be down-regulated in the apex, it can be expected that the reduced activity was at least partially the outcome of reduced transcription. Even though the activity of cwINV records only for a one-digit share of total invertase activity, from our results a lower total *in vivo* activity of invertases can be expected at the reduced temperature. Invertases supply hexoses by cleavage of sucrose when there is a high demand for it. Gibeaut et al. (1990) hypothesized from their studies that an osmotic potential gradient caused by an increased invertase activity acts as driving growth factor. Based on this hypothesis, a reduced *in vivo* invertase activity might be one reason for the reduced levels of monosaccharides in the apex in the long-term and the reduced growth of the chilling-sensitive petunia cultivar.

Considering the magnitude of carbohydrate responses to the mild chilling (**Figures 1** and **2**), we assume that also other enzymes are involved in the observed altered carbohydrate homeostasis. Studying cold acclimation in cabbage seedlings, Sasaki et al. (2001) found that enhancement of sucrose synthase and sucrose phosphate synthase activities was related to the increase of hexoses and sucrose. However, in the current gene expression analysis, genes coding for both enzymes were not significantly regulated. In general, the strong response of carbohydrate levels was not associated with a corresponding picture at transcriptional level. While the microarray data reveals a disturbance of the photosynthetic machinery at transcriptional level, it does not explain the carbohydrate accumulation particularly in the source tissue. Therefore, we hypothesize that many of the changes we observed in carbohydrate levels, were influenced by post-transcriptional enzymatic regulations. In addition, enzyme-independent transport of carbohydrates might also be important. Considering that chilling tolerance of maize was correlated to a more efficient export of assimilates from the leaves, Sowiński et al. (2001) stressed that the symplastic route of phloem loading, which is particularly susceptible to low temperature, could play an important role for a high chilling sensitivity.

### Mild Chilling Stress Inhibited Long-Term Accumulation of IAA in the Apex, Repressed JA-Signaling, and Stimulated the Ethylene Pathway in an Organ-Specific Manner

Phytohormones are known to be involved in reactions of plants to biotic and abiotic stresses. In rice, JA contents were increased and JA biosynthesis and signaling were induced under cold stress (Du et al., 2013a). In *Arabidopsis*, JA regulates cold acclimation-induced freezing tolerance (Hu et al., 2013). The low JA levels in the present study do not support a role of JA homeostasis in the growth response of the sensitive petunia cultivar to mild chilling stress. However, chilling significantly induced the expression of a jasmonate-ZIM-domain protein, which acts as repressor of jasmonate signaling. Thus, both the missing response of JA levels together with a reduced JA sensitivity may have contributed to the observed growth depression. The results of the presented study further suggest that the homeostasis of IAA and also of ABA are chilling-sensitive factors that may be involved in the growth reaction of petunia to sub-optimal temperatures. According to our results, only transient increases of ABA levels were detected during chilling stress in maize and wheat (Galiba et al., 1993; Anderson et al., 1994; Veisz et al., 1996). However, considering that ABA is obviously involved in acclimation-induced chilling tolerance in maize seedlings (Anderson et al., 1994) and in chilling tolerance of tomato (Ntatsi et al., 2013), the observed lower ABA levels in the growing tissue of shoot apices of petunia plants after prolonged chilling (**Figure 4A**) may be involved in the growth depression. Interestingly, the microarray data does not point toward a regulation of ABA at transcriptional level in the investigated tissues. This may be based on the high proportion of regulated genes without annotated protein function or may indicate that the ABA level in the apex is controlled by biosynthesis in other tissues such as roots (Ntatsi et al., 2013). The transcriptome data does also not provide a clear picture, whether the observed lower IAA level in shoot apices after prolonged exposure to chilling temperatures reflects changed auxin biosynthesis, metabolism, conjugation or transport. Nevertheless, the lower IAA concentrations may have contributed to the growth depression. IAA has a generally important function in the control of cell division, elongation, and growth (Ljung, 2013) and Rahman (2013) emphasized that cold temperatures may strongly change intracellular auxin gradients and reduce intracellular cycling thereby inducing changes in plant growth.

Considering the protective roles of both, ABA biosynthesis and reduced ethylene-signaling in tolerance of tomato against moderately sub-optimal temperature stress, Ntatsi et al. (2013) suggested that the protective role of ABA may be based on indirect control of ethylene action as it was described for conditions of water deficit stress (Sharp et al., 2000; Sharp and Le Noble, 2002). Interestingly, in the present study the expression of several genes related to the group “Ethylene biosynthesis metabolism and signaling” were affected on a long-term by chilling treatment in a tissue-dependent manner (**Figures 6** and **7**). While up-regulation of several genes in the source tissue and the internode indicate a stimulation of ethylene action, most of these genes were simultaneously down-regulated in the apex. Affected by this pattern were genes, that code for aminocyclopropane-1-carboxylate oxidase/∼4 as well as one coding for aminocyclopropane-1-carboxylate synthase, which catalyze the two steps from the precursor *S*-adenosyl-methionine over 1-aminocyclopropane-1-carboxylic acid to ethylene. In *Arabidopsis* seedlings, ethylene biosynthesis was reported to decrease in response to cold stress (Shi et al., 2012). Furthermore, Shi et al. (2012) demonstrated that the tolerance response to freezing stress is negatively regulated by ethylene. Thus, they reduced the freezing tolerance of *Arabidopsis* by inducing an overproduction of ethylene or application of the precursor 1-aminocyclopropane-1-carboxylic acid. Considering these relationships, the chilling-induced growth depression in petunia may also be mediated by ethylene action, possibly in dependence on ABA.

### Mild Chilling Stress Caused Phase-Specific and Organ-Specific Changes in Expression of Regulative Components Controlling Stress Response, DNA Replication, and Maintenance

Beyond the changes discussed above, the microarray data reflects a comprehensive phase- and organ-specific transcriptional response of the sensitive *P. hybrida* cultivar ‘Williams’ to the mild chilling (**Figures 5** and **6**). In accordance to the carbohydrate data, the opposing responses of gene expression in the two tissues during the late phase of chilling may provide indication why growth might have been reduced at the sub-optimal temperatures. On the long term, the plants seemed to attempt to up-regulate stress and DNA metabolism related processes in the source tissue in order to maintain metabolic processes and to adapt to the sub-optimal temperature On the other hand, stress stimuli related genes in the meristematic tissue of the apex and thus, in newly developing leaves were down-regulated. That might have been contributed to saving energy and to channeling resources toward coping with the imposed stress. Interestingly, the functional group “abiotic stimuli” showed a fast response, whereas on the long-term the group “biotic stimuli” was down-regulated. The acute down-regulation of genes related to abiotic stimuli in the developing meristematic tissue probably indicates a higher susceptibility to chilling stress compared with the source tissue. On the other hand, the strong up-regulation of genes related to biotic stimuli in the source leaves and in the internode in the long-term phase, which contrasted to the simultaneous down-regulation of most genes in the apex, might contribute to enhance the tolerance of the source leaves toward the continuing chilling stress. Interestingly, one expansin gene was constitutively up-regulated in the source tissue at all the time points (**Figure 7**). While expansin has primarily a growth promoting function, Goh et al. (2012) supposed its importance for counteracting growth-repressing activities in developed leaves. Considering this hypothesis, expansin seems to act here more as counterbalancing agent against the growth-depressing effects of chilling exposure than as a mere growth promotor.

The transcriptome data reflects a chilling-induced phase- and organ-specific regulation of diverse genes, which control DNA replication and maintenance as well as TFs involved in stress signaling. In the long-term phase, genes coding for different histones, as well as two genes coding for one chromomethylase, similar to chromomethylase 3 from *Arabidopsis*, were over-expressed only in the source tissue. Histones, the core elements of nucleosomes, are *S*-phase dependently expressed, when more DNA is needed. Chromomethylase 3, which is also associated with nucleosomes, has been suggested to methylate nucleosome-bound DNA. Because in *Arabidopsis* chromomethylase 3 is primarily expressed in dividing cells, it is supposed to methylate DNA during replication. However, chromomethylase 3 has also a *de novo* activity on unmethylated DNA, which points toward a maintenance function (Du et al., 2013b). Thus, the over-expression of both, histone genes and chomomethylase genes indicates a demand for more DNA, respectively, regulation of DNA, possibly caused by an induction of cell division or more likely by an induction of repair processes. A Jumonji (JmjC) domain-containing protein was one of the most constitutively up-regulated genes. Acting as histone demethylase in *Arabidopsis*, JmjC domain-containing proteins protect euchromatin from heterochromatisation. Thus, these proteins are involved in chromatin remodeling, and they further seem to function as circadian clock component (Lu and Tobin, 2011). According to these functions, the higher expression of JmjC may have contributed to the up-regulation of other genes.

Li et al. (2015) analyzed the cold stress response of cold-tolerant petunia seedlings at low temperature (2°C). They supposed that in petunia, additionally to the CBF-pathway, different other signaling systems are cross-linked to regulate the cold stress response. As potential regulators, they found cold responsive TFs especially of the TF families AP2-EREBP, GRAS, MADS-box, MYB/MYB-related, NAC, and zinc finger (Li et al., 2015). Under our conditions of mild chilling stress, applied to a sensitive petunia cultivar, we also found differentially regulated TFs, especially of the families bHLH, bZIP, ethylene responsive, homeobox, MADS-box, MYB, WRKY, and zinc finger. However, the differential regulation of genes coding for TFs was not stable over the different time points. The above-indicated ICE1–CBF transcriptional cascade has repeatedly been discussed to play an important role in reactions to chilling and cold exposure (Usadel et al., 2008; Zhang et al., 2012; Hu et al., 2013). Interestingly, the results of our microarray analysis did not provide any indication that expression of CBF genes was affected by the mild chilling. On the one hand, there is a chance that some of these genes were not recognized because they are not annotated yet. On the other hand, a lacking induction of CBF family could be related to the chilling sensitivity of the cultivar ‘Williams,’ since genes of the CBF family are expected to contribute to chilling tolerance. At least the up-regulation of JAZ in the source and the internode at 21 DoT could be an explanation for an absent CBF reaction. Hu et al. (2013) described that the cold induction of CBF/DREB1 signaling pathway itself seems to be up-regulated by jasmonate. JAZ proteins function as repressors of jasmonate signaling and interact physically with the ICE1 and ICE2 TFs. Thus, the transcriptional function of ICE1 is repressed and so the expression of its regulon weakened (Hu et al., 2013). The authors found in *Arabidopsis* that an overexpression of JAZ1 and JAZ4 repressed the freezing stress response. In addition, Zhang et al. (2012) found, by comparing distinct tolerant rice cultivars, a considerably higher recovery capacity of the tolerant cultivar at the transcriptional level.

## Conclusion

**FIGURE 9:**
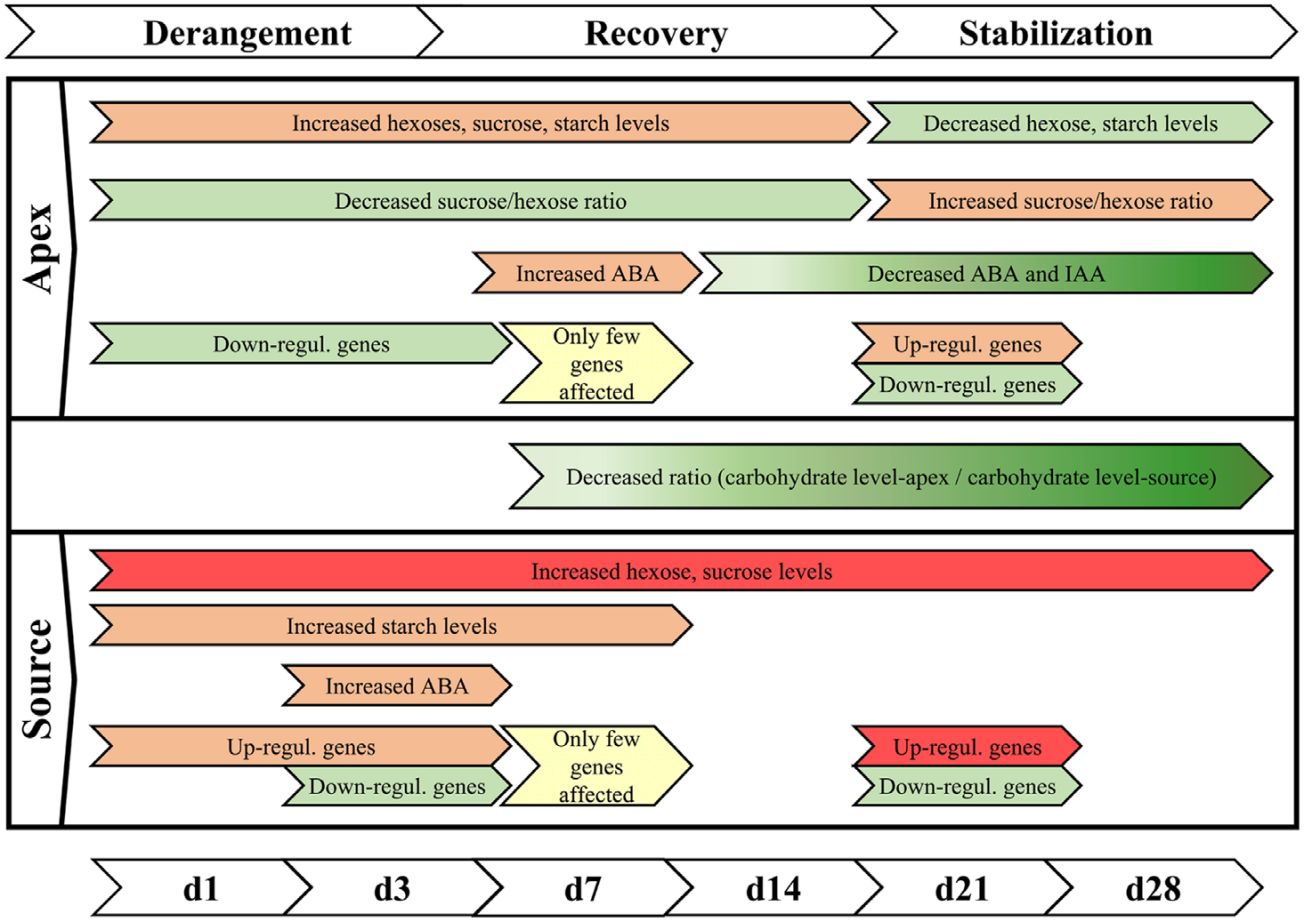
**Summary of phases of chilling reaction.** The reaction of the sensitive cultivar ‘Williams’ to the exposure to sub-optimal temperature can be characterized by three consecutive steps of derangement, recovery, and stabilization. In this context, decrease is understood as concentrations that were relative lower compared to control temperature conditions. (Red color indicates increase, green color indicates reduction, while intensity is marked by the intensity of colors; yellow indicates partial alignment to the control plants).

Even a mild chilling stress, realized with a temperature reduction of only 4 K, leads to a complex disturbance of plant functional integrity of a chilling-sensitive petunia cultivar, detectable at the levels of carbohydrates, phytohormones, and gene expression. The data as a whole reveals a holistic stress response: under chilling, specific functional units of the plant are readjusted and fine-tuned in different directions, so that the whole plant enhances the chance to survive at the expense of growth. Taking in account all presented data, a response model is proposed for a chilling-sensitive cultivar, which comprises three consecutive phases (summarized in **Figure 9**). Immediately after the reduction of temperature, the homeostasis of the plant is deranged, marked by accumulation of hexoses, and up-regulation of genes in the source tissue within the first day and an additional down-regulation at 3 DoT, while in the apex genes are mainly down-regulated on both dates. This phase of destabilization is followed by a transient phase of recovery, characterized by a trend of metabolic values toward a state similar to the plants cultivated at optimal cultivating temperature and a low number of differentially expressed genes. This phase extends into the stabilization phase, which indicates a long-term acclimation to the sub-optimal temperature. It is characterized by lower levels of hexoses, ABA and IAA and a lower sucrose/hexose ratio in the apex and lower apex/source ratios of sugars compared to the control culture. It is further marked by a high number of up-regulated genes particularly of the categories ‘Biotic Stimuli’ and ‘Ethylene metabolism and Perception’ in the source tissue, whereas genes of the category ‘Biotic Stimuli’ are down-regulated in the apex. This late response obviously helps the plant to tolerate the sub-optimal temperature without damage but at the expense of reduced growth. Future experiments will focus on the question, whether cultivars differing in the growth response to mild chilling exhibit tolerance-specific response profiles at the molecular physiological levels described.

## Conflict of Interest Statement

The authors declare that the research was conducted in the absence of any commercial or financial relationships that could be construed as a potential conflict of interest.

## Footnotes

1 www.statsoft.com

2 http://strep-microarray.sbs.surrey.ac.uk/RankProducts/

3 http://caps.ncbs.res.in/stifdb2/index.html
