# Supplementary material for: Transcriptome, carbohydrate, and phytohormone analysis of Petunia hybrida reveals a complex disturbance of plant functional integrity under mild chilling stress
Source: Front Plant Sci. 2015 Jul 28;6:583. doi: 10.3389/fpls.2015.00583 (PMC4517316; doi:10.3389/fpls.2015.00583)
Supplement: Supplementary file 2 [file Presentation_2.PDF]

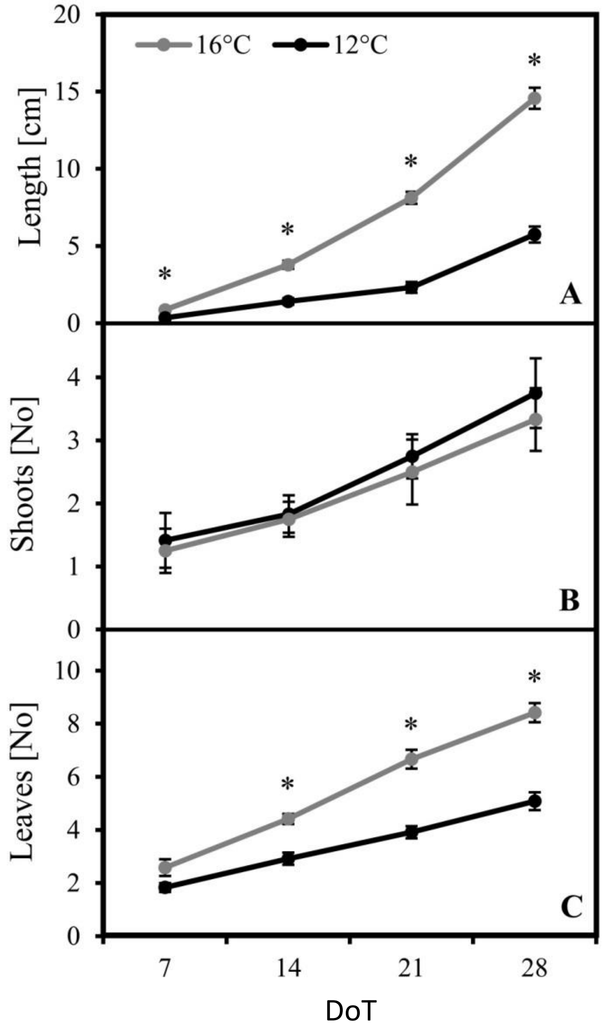

Figure S2│Growth parameters. Impact of sub-optimal temperature (black: 12.1°C (day/night: 13.1°C/11.1°C), grey: 16.0°C (16.8°C/15.2°C)) on different growth parameters over the period of 28 days after differentiation of temperature (DoT). Shown are increases compared to 0 DoT. (A) length increase of the main shoot; (B) number of newly developed shoots; (C) number of newly developed leaves on the main shoot. (greenhouse; n=12; data are means +/- SE; asterisks indicate significant differences between temperature treatments for a given sampling time, $P \leq 0.05$).
